# Genomics of Human Fibrotic Diseases: Disordered Wound Healing Response

**DOI:** 10.3390/ijms21228590

**Published:** 2020-11-14

**Authors:** Rivka C. Stone, Vivien Chen, Jamie Burgess, Sukhmani Pannu, Marjana Tomic-Canic

**Affiliations:** 1 Wound Healing and Regenerative Medicine Research Program, Dr. Phillip Frost Department of Dermatology and Cutaneous Surgery, University of Miami-Miller School of Medicine, Miami, FL 33136, USA; vivienyc@med.miami.edu (V.C.); jlb452@med.miami.edu (J.B.); 2 Medical Scientist Training Program in Biomedical Sciences, University of Miami Miller School of Medicine, Miami, FL 33136, USA; 3 Department of Dermatology, Tufts Medical Center, Boston, MA 02116, USA; drsukhmanipannu@gmail.com; 4 John P. Hussman Institute for Human Genomics, University of Miami-Miller School of Medicine, Miami, FL 33136, USA

**Keywords:** fibrosis, genomics, genome-wide association study, transcriptome, wound healing, keloid, scleroderma, cirrhosis, pulmonary fibrosis, chronic kidney disease

## Abstract

Fibrotic disease, which is implicated in almost half of all deaths worldwide, is the result of an uncontrolled wound healing response to injury in which tissue is replaced by deposition of excess extracellular matrix, leading to fibrosis and loss of organ function. A plethora of genome-wide association studies, microarrays, exome sequencing studies, DNA methylation arrays, next-generation sequencing, and profiling of noncoding RNAs have been performed in patient-derived fibrotic tissue, with the shared goal of utilizing genomics to identify the transcriptional networks and biological pathways underlying the development of fibrotic diseases. In this review, we discuss fibrosing disorders of the skin, liver, kidney, lung, and heart, systematically (1) characterizing the initial acute injury that drives unresolved inflammation, (2) identifying genomic studies that have defined the pathologic gene changes leading to excess matrix deposition and fibrogenesis, and (3) summarizing therapies targeting pro-fibrotic genes and networks identified in the genomic studies. Ultimately, successful bench-to-bedside translation of observations from genomic studies will result in the development of novel anti-fibrotic therapeutics that improve functional quality of life for patients and decrease mortality from fibrotic diseases.

## 1. Introduction

### 1.1. Background and Scope

Fibrotic disorders are implicated in nearly 45% of all deaths in the developed world [1]. Pathological fibrogenesis is associated with an uncontrolled wound healing response and is remarkably similar at the cellular level across different organs. Classically, disease-specific triggers initiate site-specific injuries, which activate distinct cells that drive fibrosis in genetically susceptible individuals. More specifically, an acute injury becomes a persistent inflammatory stimulus that sustains the production of fibrogenic growth factors, proteolytic enzymes, and cytokines, which collectively stimulate excess extracellular matrix (ECM) deposition and remodeling, destroying tissue architecture and resulting in progressive functional impairment and sometimes death [2,3,4] (Table 1). Intriguingly, fibrotic damage may be reversible with the removal of the inciting inflammatory trigger [5], yet there is a dearth of effective treatment strategies targeting fibrosis.

In this review, we summarize genomic studies designed elucidate the injury-driven pathogenesis of human fibrosing disorders of the skin, liver, kidney, lung, and heart. These encompass genome-wide association studies (GWAS) identifying loci that confer susceptibility to fibrotic disease; transcriptome analyses (microarray, whole exome and RNA-sequencing) identifying differential gene expression changes in lesional fibrotic tissue and ex vivo fibroblasts; profiling of epigenomic factors, specifically non-coding RNA (microRNA (miRNA), circular RNA (circRNA), and long non-coding RNA (lncRNA)); and DNA methylation studies that define global regulatory networks of fibrosis. In each organ, we describe the initial acute injury that drives unresolved inflammation, identify genomic studies that have defined the pathogenic gene changes leading to excess matrix deposition and fibrosis, and conclude with a summary of therapies directly targeting pro-fibrotic factors as well as removing the inappropriate wounding response to reverse existing fibrosis. We restrict the scope of this review to genomic studies in humans, i.e., those performed on biomaterials from patients with fibrosing disorders. Ultimately, successful translation of genomic findings to the development of targeted therapeutics will result in decreased morbidity and mortality from fibrotic diseases.

### 1.2. Inflammation, Remodeling and Fibrosis in Wound Healing

The wound healing process features overlapping phases of hemostasis, inflammation, proliferation, and extracellular matrix (ECM) remodeling that are tightly regulated to prevent damage and, in skin, quickly restore epidermal barrier [6]. Inflammatory response during wound healing is generated by complex interplay between systemic and resident immune cells, and tissue-specific cell types and is a main trigger for post-injury scar in adult organism [6]. Inflammation during acute wound injury activates the transition of fibroblasts to myofibroblasts to release ECM proteins that remodel the surrounding tissue [7]. Of mesenchymal origin, fibroblasts express vimentin and contain a large volume of endoplasmic reticulum and golgi to facilitate the excretion of extracellular matrix proteins such as collagens, proteoglycans, and fibronectin. Activated myofibroblasts additionally have α-smooth muscle actin stress fibers, with increased fibronectin and gap junction expression, and lack desmin and myosin smooth muscle markers [7]. Resident epithelial cells also shift to myofibroblast phenotype through an epithelial mesenchymal transition (EMT) process [8]. Early and prolonged EMT activation promotes excessive inflammation and fibrogenesis (reviewed in [8]). Regardless of the tissue type ECM, including its disruption by injury, its production or its remodeling, provides biomechanical forces that contribute to overall mechanisms of scar formation [9].

While myofibroblasts are primary players in remodeling, the inflammatory wounding response also features immune cells that signal and release profibrotic mediators that contribute to ECM deposition and fibrosis (reviewed in [10]). For instance, neutrophils release neutrophil extracellular traps (NETs) that further activate fibroblasts and promote differentiation into myofibroblasts [11].

Dysregulation of these processes in any connective tissue-containing organ can cause excessive ECM deposition and tissue fibrosis, ultimately leading to loss of organ function [6]. As an example, robust yet short-lived wound healing response of injured oral epithelium can result in scarless healing [12], while a prolonged response with pro-fibrotic mediators can produce the non-healing bed of chronic wounds [8,13].

## 2. Fibrosing Disorders of the Skin

Physiologic wound healing after skin injury is a dynamic process aimed at efficiently restoring a compromised epidermal barrier, and proceeds through distinct yet overlapping phases of inflammation, proliferation, and remodeling. The inflammatory response drives repair and is tightly regulated in a temporal fashion to minimize damage such as excessive scarring. While fetal skin is capable of scarless regeneration [14], postnatal skin injury results the formation of a scar, and a sustained remodeling response leads to deposition of excess ECM and fibrotic scarring with loss of structure and function [6]. Fibrosing skin disorders encompass hypertrophic scars and keloids, a spectrum of localized and diffuse sclerodermoid conditions, and chronic (non-healing) skin ulcers with fibrotic wound beds.

### 2.1. Keloids and Hypertrophic Scars

Hypertrophic scars and keloids are induced by skin injury deep enough to affect the reticular dermis that involves excess collagen production during the wound healing process [15,16]. Hypertrophic scars typically develop after surgery or trauma such as burns, and appear as thick raised lesions characteristically contained within the site of injury that may regress spontaneously [6]. In contrast, keloids extend beyond the original borders of the injury, display horizontal growth without regression, and have a higher recurrence rate after excision [15]. Histologically, the collagen in hypertrophic scars is arranged in a regular pattern parallel to the dermis, whereas keloids feature irregularly arranged collagen and an increased ratio of type I: type III collagen [15].

Population-level genomic studies have played a key role in elucidating the pathogenesis underlying abnormal scar formation in the skin. Both family history and darker skin color are associated with increased incidence of keloids [17], suggesting a genetic predisposition, though no single causal gene has been implicated, and a non-mendelian polygenic pattern of inheritance is most likely [15,16]. A multistage GWAS of keloid predisposition in a Japanese population identified several single nucleotide polymorphisms (SNPs; rs873549, rs1511412, rs940187, and rs8032158) in three chromosomal regions in linkage disequilibrium with SNPs in FOXL2 and NEDD4 [18]. 3 of these SNPs were subsequently confirmed in a Chinese Han population with further identification of risk and protective keloid haplotypes [19], and additional genomic studies further correlated rs8032158 with clinical keloid severity [20]. In African Americans, an approach using admixture mapping and whole exome association identified SNPs in two myosin genes, MYO1E and MYO7, as being associated with keloid formation [21]. GWAS of Japanese and African American families displaying autosomal dominant keloid inheritance identified susceptibility loci on chromosomes 2q23 and 7p11, respectively [22]. Notably, unique SNPs located within NEDD4 (chr15q21.2–22.3) independently showed association with keloid risk in multiple populations [21].

Genomic studies have profiled hypertrophic scars and keloids utilizing full-thickness skin biopsies, laser capture-microdissected segments, and patient tissue-derived fibroblasts cultured ex vivo, as the sources of genomic material [23]. Gene expression profiles of fibroblasts from progressing vs. regressing sites within individual keloid lesions have also been compared [24]. Upregulated genes in keloids include Interleukin (IL)-1α, IL-1β, IL-6, tumor necrosis factor-α [15,25]; insulin-like growth factor-binding proteins (IGFBP)-3, -5, -7 and connective tissue growth factor (CTGF; also associated with fibrosis in systemic sclerosis (SSc), idiopathic pulmonary fibrosis (IPF) and leiomyomas) [26]; chondrocyte and osteoblast differentiation markers such as RUNX2 (in both African American and Japanese populations); and associated induction of transcription factors SOX9 and scleraxis and upstream factors Trichostatin A and CAV1 [27,28,29]. HIF-1α is upregulated in keloid fibroblasts, and HIF-1α inhibition reduces collagen levels in keloid as well as hypertrophic scar fibroblasts [30,31] introducing a pathophysiological link between a hypoxic microenvironment and collagen production in keloids and hypertrophic scars. FKBP10 is upregulated in hypertrophic scars, regulating α-SMA expression and pro-collagen maturation in fibroblasts; it may represent a novel therapeutic target [32]. Wnt pathway inhibitors and several IL1-inducible genes were decreased in fibroblasts cultured from keloids in comparison with normal scars [26]. At the pathway level, aberrant fibroblast activation in conjunction with altered transforming growth factor beta (TGF-β)-related signaling has been observed [15]. Moreover, integrative RNA-seq and microRNA expression analysis of keloid formation in keloid-prone Taiwanese patients [33] identified temporal changes in the initiation, progression, and maintenance of keloids; this study noted decreased Notch signaling and Toll-like receptor pathway activity as well as mitogen-activated protein (MAP) kinase signaling, which remained dysregulated six weeks after wounding.

Epigenetic factors such as non-coding RNA (miRNA, circRNA, lncRNA) and DNA methylation also modulate genomic changes in keloids and hypertrophic scars. Analysis of miRNA expression patterns in keloids reveal decreased miR-194-3p, an inhibitor of fibroblast proliferation and migration [34] and decreased miR-199a-5p [35], a fibrotic regulator also associated with IPF and SSc. Downregulation of miR-196a, which binds COL1A1 and COL3A1, has been associated with an increase in secreted type I and III collagen in keloids [36]. Additional differentially expressed miRNA have been identified and connected to target genes that are involved in MAP kinase and HIF-1 signaling pathways [37]. In hypertrophic scars, downregulation of miR-200b was associated with increased cell proliferation, decreased cell apoptosis, and altered collagen I and III production of fibroblasts in vitro [38]. Further upstream, a series of circRNAs are upregulated in keloid tissue, interacting with fibrosis-associated miR-29a, miR-23a-5p, and miR-1976 to form a regulatory network with predicted role in keloid development [39].

Microarray profiling of long noncoding RNAs (lncRNAs) has also been performed in lesional keloid tissue, identifying altered expression of CACNA1G-AS1 and HNF1A-AS1, which interact not only with pro-fibrotic TGF-β pathways, but also with cellular adhesion and tight junction signaling [40,41]. Another study reported altered expressions of 30 lncRNAs relating to hedgehog signaling pathways, which were accompanied by mRNA changes reflecting upregulation of cell proliferation and tissue repair and downregulation of apoptotic pathways in keloid tissue [42]. Similarly, lncRNAs NR_125715 and NR_046402 differentially expressed in fibroblasts from hypertrophic scars correlated with expression levels of TGF-β pathway genes TGFB2 and POLD1 transcripts [43]. Finally, genome-wide profiling of DNA methylation in keloids and normal skin identified a series of differentially hyper- and hypo-methylated genes in keloids with roles in diverse signaling pathways, providing new opportunities for the study of keloid pathogenesis [44]. In addition, hypermethylation of the SFRP1 promoter has been shown to contribute to downregulated Wnt/beta catenin signaling in keloids [45].

Currently, there are a range of treatment options for keloids and hypertrophic scars, including topical and intralesional corticosteroids, radiotherapy, compression therapy, 5-fluorouracil therapy, and surgical approaches to reduce tension [15,16]. However, although genomic studies have identified many promising targets in the fibrotic cascade [46], successful translation to targeted therapies in the clinical setting has yet to occur.

### 2.2. Scleroderma (Systemic Sclerosis, Localized Scleroderma, and Lichen Sclerosus)

Scleroderma, or “hardened skin”, is a condition of autoimmune fibrosis that is implicated in a spectrum of diseases including systemic sclerosis (SSc), localized scleroderma, and lichen sclerosus. Systemic sclerosis (SSc) is a rare autoimmune connective tissue disease that presents with symmetric hardening of the skin of the fingers, hands, and face (“limited cutaneous SSc”) that may spread to other sites (“diffuse cutaneous SSc”); both cutaneous SSc subtypes may involve fibrosis of visceral organs. Localized scleroderma (LSc), sometimes termed morphea, is an inflammatory disease of the dermis and subcutaneous fat that leads to scar-like fibrosis, while lichen sclerosus primarily involves atrophic, fibrotic skin lesions most commonly occurring in genital and perianal areas and affecting the epidermis and superficial dermis [47,48]. Both LSc and lichen sclerosus are not associated with systemic fibrosis. The pathological mechanism behind systemic and localized forms of scleroderma is hypothesized to be microvascular injury. Endothelial cell injury promotes perivascular injury and autoimmune reactivity, inducing fibroblast activation, tissue fibrosis, and eventually scleroderma-like disease [49], though etiology of the initial endothelial injury is unclear [2]. In addition, uncontrolled NETosis occurs in SSc neutrophils and contributes to early progression of fibrosis [50].

Genomic studies have furthered understanding of fibrotic mechanisms in sclerodermoid disorders, with SSc as the prototypical condition. GWAS have identified susceptibility loci for SSc in extracellular matrix genes (COL4A3, COL4A4, COL5A2, COL22A1, COL13A1, CTGF) and autophagy (ATG5) as well as genes involved in (auto)immunity: interferon signaling (IRF4, IRF5, STAT4), interleukin signaling (IL12A, IL12RB1, IRAK1), and B-cell signaling (BANK1) (reviewed in [2,51]). As such, an exacerbated immune response to endothelial cell injury in genetically prone individuals might provoke a sustained autoimmunity that leads to fibrosis [2]. Additional GWAS coupled with Immunochip array data investigating alleles, amino acid residues, and SNPs across the human leukocyte antigen (HLA) region identified polymorphisms particularly in class II HLA genes that show significant association with SSc susceptibility [52,53].

Gene expression profiling of the skin from patients with SSc have identified distinct gene signatures that correlate with extent of skin fibrosis and end-organ damage. One study [54] identified distinct keratin versus fibroinflammatory signatures, in which a predominating keratin pattern correlated with shorter disease duration and presence of interstitial lung disease, while the fibroinflammatory pattern correlated with diffuse cutaneous involvement and greater skin thickening. Other studies have utilized gene expression patters to further divide cutaneous SSc into inflammatory, fibroproliferative, limited, and normal-like [48,55,56,57,58], wherein the inflammatory subset involves infiltrating T cells, B cells, and macrophages, with the profibrotic IL-13–IL-4 pathway primarily driving fibrosis [56,57]; NOTCH4, IRF7, and GRB10 are differentially expressed in this subset [48]. The fibroproliferative subset involves a pro-fibrotic platelet-derived growth factor (PDGF) pathway, though TGF-β signaling is involved in both inflammatory and fibroproliferative subsets. Limited and normal-like subsets are comprised of profiles reflecting a non-proliferative, immunologically quiescent state [56]. In another study, datasets of SSc patients were integrated and analyzed to identify a common 415-gene signature that accurately distinguished SSc patients from healthy controls [59]. Integrative multi-tissue network analysis [60] also used multiple datasets to identify a gene expression signature involving pro-fibrotic macrophages that were common among various SSc-affected tissues (skin, lung, esophagus, and peripheral blood), supporting the presence of fundamental mechanisms driving fibrosis across organs in SSc.

A series of miRNA expression profiling studies have identified miRNAs involved in fibrosis and ECM formation in scleroderma, and several miRNAs have demonstrated utility as biomarkers of disease activity and severity. One of the most extensively studied is anti-fibrotic miRNA-29, which is strongly downregulated in SSc fibroblasts as compared to healthy controls and is and associated with increased mRNA and protein expression of types I and III collagen [61,62]. Exogenous stimulation with known SSc pro-fibrotic mediators TGF-β, PDGF-B, and IL-4 decreased miR-29a expression in skin fibroblasts [61], suggesting downstream involvement of miR-29a in these pathways as well. In contrast, skin from patients with localized scleroderma did not demonstrate downregulation of miR-29a, instead featuring downregulation of miR-7, which correlated with COL1A2 overexpression [63]. In both SSc and LSc tissues, downregulation of let-7a compared to normal and keloid skin was associated with excess type I collagen expression [64], and upregulation of miR-483-5p was associated with increased type IV collagen expression [65]. Other fibrosis-associated miRNAs in SSc include downregulated antifibrotic miR-150 [66] and miR196a [36] (also decreased in keloids), and upregulated profibrotic miR-21, miR-92a, miR-145, and miR-202-3p [62,67].

lncRNA function in fibrosis of SSc patients is has also been explored. lncRNA TSIX is increased in serum and skin fibroblasts of patients with SSc compared to SLE and healthy patients, involving in type I collagen regulation and the TGF-β signaling pathway [68]. RNA sequencing of SSc skin biopsy samples have also further identified a group of antisense lncRNAs, primarily associated with significant deregulation of genes CTBP1, OTUD6B, and AGAP2 [69].

Treatment options for SSc include systemic immunosuppressants (e.g., mycophenolate mofetil, cyclophosphamide, methotrexate, and anti-IL6 receptor antibody, tocilizumab) to dampen autoinflammation. Targeted therapies based upon findings from genomic studies are also becoming available; for instance, clinical trials have been performed for abatacept (targeting CTLA4-associated inflammation) and romikimab (targeting IL-4 and IL-13-mediated fibrosis) [2]. An open-label trial of fresolimumab, a high-affinity antibody targeting all three TGF-β isoforms (TGF-β1, TGF-β2, and TGF-β3), successfully inhibited TGF-β signaling in SSc, as demonstrated by decreased mRNA of TGFβ regulated genes, decreased myofibroblast infiltration, and decreased skin fibrosis [70]. Moreover, the genomic signatures that are used to classify patients into subsets as discussed above may have therapeutic implications, as it was observed that patients with the inflammatory subset improved more significantly when treated with mycophenolate mofetil [55] and abatacept [71] than those with fibroproliferative and normal-like subsets. That said, there is debate over whether the genomic signatures change or reflect disease progression within the individual [48,58], and further studies on disease course and clinical presentation in relation to gene expression changes are needed.

### 2.3. Chronic Ulcers

Chronic non-healing ulcers (diabetic foot ulcers, venous leg ulcers, and pressure ulcers) are a widespread clinical challenge, affecting up to 2% of the population, and are associated with impaired quality of life, chronic pain, physical disability, and increased mortality in patients [72]. Fibrosis is a shared histologic feature that is identified in approximately 50% of chronic wounds and aids in guiding the margin of therapeutic debridement [73]. In non-healing ulcers, an inappropriately prolonged acute inflammatory response transitions to chronic ineffective inflammation that fails to result in re-epithelialization and is associated with a sustained remodeling response and the development of a fibrotic ulcer bed [6]. In venous leg ulcers (VLU) in particular, the wound bed is histopathologically characterized by disorganized ECM, marked fibrosis, and chronic inflammatory infiltrates, all of which contribute to impaired healing; in fact, increased presence of dense fibrosis and high mature collagen levels in VLUs correlate with poor healing outcomes in the clinical setting [74]. Using microarray profiling, our group reported enrichment of inflammatory response and fibrogenetic pathways in non-healing VLU [13], corresponding to the histological findings of disorganized ECM, fibrosis, and chronic inflammation [73,74,75]. Specifically, we observed enrichment of collagens and secreted matricellular proteins in conjunction with upregulation of fibronectin (FN1), tenascin (TNC), osteopontin (SPP1), connective tissue and hepatocyte growth factors (CTGF, HGF), and PAI-1 (SERPINE1). Pro-fibrotic canonical TGF-ß signaling was highly enriched and activated in the lesional VLU bed as well, thus marking the chronic VLU as a fibrotic skin disease. We extended our findings in a randomized controlled post-marketing clinical trial examining the mechanism of action of a bioengineered bilayered cellular construct (BLCC), a commercial skin substitute with demonstrated efficacy in promoting VLU closure. BLCC application triggered an acute wound healing response at the ulcer edge to reverse chronic inflammation [76] while coordinately stimulating remodeling of the ulcer bed, as evidenced by decreased expression of profibrotic TGFß1 gene targets, increased levels of TGF-ß inhibitor decorin, and endogenous release of matrix metalloproteinase (MMP)-activating zinc to stimulate antifibrotic remodeling [13]. As such, the development and application of anti-fibrotic therapies represent a novel treatment approach for VLUs and other non-healing ulcers.

### 2.4. Exogenous Triggers

Other instances of pathological skin fibrosis occur in burn wounds and radiation induced injury, and genomic studies have helped identify additional signaling factors and pathways involved. Healing of burns can involve extensive hypertrophic scarring and contracture in up to 60% of wounds [77]. GWAS in adults with deep-to-partial-thickness burns have associated reduced severity of hypertrophic scarring with intronic variants in CSMD1, which encodes proteins involved in regulation classical and lectin complement pathways, neuronal growth and tumorigenesis, and with PTPN5, a MAPK inhibitor expressed in neurons [78,79]. A polymorphism in melanocortin-1 receptor (MC1R) is associated with increased risk for severe hypertrophic scarring via increased fibroblast proliferation [79]. An exome-wide array association study with gene pathway analysis also associated genes involving innervation and cell adhesion with scar height and scar pliability of patients with burn injuries; however, the study failed to replicate findings of significantly associated SNP variants previously described [80]. Microarray analysis of cutaneous gene expression during the first 17 days post-burn injury revealed significant upregulation of the osteopontin (SPP1), previously shown to impede wound repair and induce inflammation-triggered fibrosis. IL6 and IL8 were also upregulated, confirming involvement of common fibrotic and inflammation pathways in the hypertrophic scarring of burn patients [77].

Radiation-induced injury is commonly defined by endpoints such as telangiectasia, atrophy and, most significantly, fibrosis that impairs function and decreases quality of life [81]. GWAS have identified polymorphisms in TGFB1 as well as XRCC1 (which encodes a protein involved in DNA base excision repair) as conferring risk for radiation-associated fibrosis. In breast cancer patients, TGFB1 −509T and +869C alleles are associated with increased risk of fibrosis [82], while XRCC1 rs2682585 has been associated with decreased risk of fibrosis post radiation therapy [83]. The association of TGFB1 −509T with increased radiation-induced fibrosis was subsequently validated in a prospective cohort study following breast cancer patients over a minimum of 3 years after whole breast irradiation [84]. Conversely, wild type alleles of TGFB1 and XRCC1 were associated with a lower grade of fibrosis post radiation therapy in head and neck cancer patients [85]. Significant association was also found in breast cancer patients between radiation-induced fibrosis and polymorphism of TXNRD2, a mitochondrial enzyme important in reactive oxygen species removal [86]. This link suggests that, in addition to common fibrotic signaling and DNA repair pathways, impaired antioxidant response plays a role in post-radiation fibrosis. Gene expression profiling of whole blood collected from breast cancer survivors post-radiotherapy identified 87 differentially expressed genes, most significantly involving downregulation of TGF-β1 signaling and interleukin-2 pathways, along with upregulation of plasminogen activator inhibitor 1, a transcriptional activator inhibiting fibrinolysis [87]. With respect to epigenetic mechanisms, DNA methylation profiling of dermal fibroblasts from breast cancer patients identified diacylglycerol kinase alpha (DGKA) as a promising region of regulation, in which hypomethylation led to EGR1-mediated induction of DGKA associated with increased risk of fibrosis [88]. Despite the contribution of genomic studies in elucidating pathogenic pro-fibrotic mechanisms, targeted therapies aimed at preventing or treating radiation-associated fibrosis are not currently available in the clinical setting.

Nephrogenic systemic fibrosis is a rare, progressive disorder of skin thickening, hyperpigmentation, and extracutaneous fibrosis that develops following administration of gadolinium-based contrast agents in a subset of patients with renal insufficiency [89,90,91]. In this condition, an exaggerated fibrogenic response features high numbers of macrophages and fibroblasts, increased circulating monocytes, and higher levels of TGF-β in dendritic cells of skin and fascia from patients [92,93]. Among the few genomic studies performed in patients with this condition, one case-control genotyping study failed to identify associations of disease incidence with polymorphisms in profibrotic mediators TGFB1 and CAV1, though further studies are needed.

## 3. Hepatic Fibrosis

Like other organs, liver fibrosis is the end product of chronic inflammation in response to injury from a wide variety of sources including alcohol, viral infection, non-alcoholic steatohepatitis, and cholestasis [94]. A majority of myofibroblasts arise from activated hepatic stellate cells (HSC), although endogenous fibroblasts, fibrocytes, bone-marrow derived cells, and liver parenchymal cells can be a source as well. Hepatic stellate cells secrete ECM proteins and metalloproteinases to remodel tissue. Hepatic fibrosis is characterized by the replacement of collagen IV and VI with collagen types I and II and fibronectin [94]. A concert of chemokine molecules, adipokines, neurotropic factors, inflammatory feedback loops, oxidative stress, macrophages, T cells, neutrophils, and mast cells contribute to fibrogenesis [95]. Cirrhosis develops with progression of hepatic fibrosis, and is characterized by abnormal hepatocyte regeneration, liver nodules and changes in vasculature [96]. Liver cirrhosis, in conjunction with predisposing genetic and environmental factors, sets the stage for hepatocellular carcinoma and terminal liver dysfunction. While fibrosis is a common denominator in liver dysfunction, hepatic disease can be generally divided into non-alcoholic fatty liver disease (NAFLD), alcoholic cirrhosis, and primary biliary cirrhosis (PBC). Notably, due to the liver’s regenerative capacity, fibrosis may sometimes be reversible upon removal of the inciting inflammatory factors, making these attractive targets for therapeutic intervention [95].

Several GWAS have been conducted in patients with NAFLD. A GWAS in a Japanese cohort identified the SNP in PNPLA3 as associated with NAFLD [97]. A 2011 GWAS of several cohorts including the AGES, Family Heart, Old Order Amish, and Framingham Heart studies also identified PNPLA3 associations as well as NCAN, GCKR, and LYPLAL1 [98]. A recent GWAS of a European cohort confirmed PNPLA3 as a risk factor for NAFLD and additionally identified TM6SF2, HSD17B13, and PYGO1 [99]. A study of patients in the electronic medical records and genomics network (eMERGE) identified 3 SNPs at the PNPLA3-SAMM50 area (rs738409, rs738408, and rs3747207) as well as IL17RA and ZFP90-CDH1 [100]. In a Hispanic pediatric male cohort, a GWAS identified SNP rs6128907 near actin related protein 5 homolog (ACTR5) and SNP rs11166927 in TRAPPC9 to be associated with fibrosis progression and NAFLD severity. [101]. A transcriptome analysis of RNA from plasma and liver biopsies in separate cohorts with NAFLD showed a statistical upregulation of osteopontin (SPP1) and CXCL10, serving as a potential biomarker of NAFLD [102]. In human samples of non-alcoholic steatohepatitis, miR-223 was elevated and implicated in the progression of steatosis to nonalcoholic steatohepatitis [103]. GWAS of non-alcoholic steatohepatitis patients treated with obeticholic acid, an activator of farnesoid X nuclear receptor, identified loci associated with successful reversal of fibrogenesis following treatment [104]. For a recent review on ‘omics of non-alcoholic fatty liver disease, see the review by Perakakis et al. [105].

While abstinence from alcohol has proven beneficial at reversing fibrosis [106], the underlying genomic changes in alcoholic cirrhosis are poorly understood. In one study, mice and human cultured HSCs were compared using single-cell RNA-seq; CSF1R, PLEK, LAPTM5, CD74, CD53, MMP9, CD14, CTSS, TYROBP, and ITGB2 were upregulated in both alcohol-induced mouse HSCs and alcohol liver disease HSCs [107]. GWAS studies of a Mestizo Mexican and European Caucasian cohorts found the SNP rs738409 in PNPLA3 to be linked with alcoholic liver disease, similar to NAFLD [108,109]. To date, however, the majority of genetic analyses in alcoholic cirrhosis have focused on alcoholism-related behavior or alcohol metabolism rather than the progression of liver damage in alcoholic cirrhosis [110].

Several GWAS studies of PBC have been performed in Japanese, North American, Italian, and other European cohorts. In Japanese cohorts, TNFSF15, POU2AF1, ARHGAP31, TMEM39A, POGLUT1, TIMMDC1, and CD80 were found to be susceptibility loci of primary biliary cholangitis [111,112], while SNP rs13720 in CTSZ was strongly associated with jaundice progression [113]. Another Japanese GWAS compared Crohn’s disease and PBC and found susceptibility loci in ICOSLG and IL12B to be associated with both disorders [114]. A GWAS of 2072 North American patients identified 16 SNPs associated with PBC with the strongest association at the HLA-DQB1 locus as well as SNPs at IL12A and IL12RB2, implying a role for interleukin-12 in the development of primary biliary cirrhosis [115]. In the same North American cohort, additional associated SNPs in STAT4 and CTLA4 loci were identified. GWAS in an Italian cohort replicated the association with IL12A and IL12RB and further identified susceptibility in SPIB, IRF5-TNPO3, and 17q12-21 loci [116]. Another European cohort including more than 7000 individuals identified 12 new loci related to biliary cirrhosis, among them were STAT4, IL7R, CD80, IKZF3, CXCR5, TNFRSF1A, and NFKB1 [117]. Finally, viral hepatitis and alpha-1 antitrypsin deficiency also contribute to the burden of fibrotic liver disease, and fibrosis severity in patients coinfected with human immunodeficiency virus (HIV) and HCV was associated with locus on chromosome 3p25 (rs61183828) neighboring CAV3 and RAD18 [118].

There has been an abundance of clinical evidence showing the regression of liver fibrosis with the removal of the inciting agent, making it the current best anti-fibrotic therapy [119,120,121]. Removal of the offending toxin decreases cytokine levels and increases collagenase and metalloproteinase activities to remove ECM [122], and there are ongoing clinical trials of therapies directed at apoptosis inhibition, HSC inhibition, and immune modulation [123]. In the phase II FLINT clinical trial, obeticholic acid showed benefit in liver fibrosis and improvement of steatosis and inflammation in patients with nonalcoholic steatohepatitis [104]. Unfortunately, preclinical-to-clinical translation of many antifibrotic therapies from mice to humans has proven challenging; while ACE inhibitors, ARBs, the insulin sensitizing agent farglitazar, the PPARγ agonist pioglitazone, and TNFα antagonist pentoxifylline showed promising pre-clinical results, clinical trials showed no significant antifibrotic benefit in humans [124,125,126,127]. Beyond identifying novel therapeutic targets, genomics can be used to create non-invasive diagnosis tools. Transcriptome analysis of peripheral blood in patients with HBV infection identified COL5A1, HLA-DQB1, MMP2, and CDK4, and created an edge-based panel for determining diagnosis and prognosis of hepatocellular carcinoma in patients [128]. As results of ongoing genomic studies in the various types of liver disease become available, new biomarkers and therapeutics will hopefully be developed.

## 4. Renal Fibrosis

### 4.1. Chronic Kidney Disease

Renal injury, inflammation, and subsequent fibrosis can be triggered by exogenous insults such as obstruction and infection. However, the majority of antifibrotic research centers on chronic kidney disease (CKD), most often resulting from diabetes and hypertension. CKD is histopathologically characterized by progressive extracellular matrix replacement of interstitial or glomerular tissue [129,130]. During CKD progression, pro-inflammatory and profibrotic cytokines and growth factors are continually released, leading to an excessive ECM accumulation and resulting in kidney fibrosis.

Beginning in 2009, GWAS have been conducted in numerous populations (East Asian, African American, Native American, Hispanic/Latino, and European cohorts) [131,132,133,134,135,136,137,138,139,140] and regions (Sri Lanka, Saudi Arabia) [141,142] to identify CKD-associated loci. In a meta-analysis of four European cohorts, CKD susceptibility variants were identified in SHROOM3 and STC1 [143], which was followed by an expanded CKDGen study that correlated renal function and creatinine indices with susceptibility loci in or near a series of genes with roles in podocyte function, angiogenesis, solute transport and renal metabolism [134,144]. Notably, many of the GWAS commonly identified various SNPs in UMOD as being strongly associated with CKD risk. UMOD encodes the protein uromodulin that is produced in the loop of Henle, and while its exact function in renal fibrosis is unclear, rare mutations in UMOD are associated with autosomal dominant kidney diseases such as familial hyperuricemic nephropathy and medullary cystic kidney disease type 2 [143] and there is evidence that uromodulin is a non-invasive urinary biomarker of nephron tubule health in CKD [145]. GWAS in patients with diabetic kidney disease/diabetic nephropathy identified a strong association with a variant in COL4A3, a structural component of the glomerular basement membrane [146]. Similar studies in a type 2 diabetes cohort reported associations with UMOD and PRKAG2, both also correlated with estimated glomerular filtration rate; an expanded analysis in larger cohorts confirmed these variants and further identified an association between GABRR1 and microalbumiuria [147]. Another GWAS [132] further associated UMOD, GALNT11, and CDH23 variants with CKD progression. A compartment-specific GWAS combined with expression of quantitative trait (GWAS-eQTL), wherein the kidney glomerulus and tubule compartments were microdissected prior to GWAS-eQTL to enhance gene identification by reducing cellular heterogeneity; identified DAB2 as a likely causal gene for CKD development [148].

Missing from GWAS studies are direct associations with TGF-β, whose canonical signaling drives renal fibrosis and whose pro-fibrotic actions are regulated by noncoding RNAs and epigenetic changes [3]. Meta-analysis of GWAS in type 1 diabetic nephropathy found that renal fibrosis is associated with AFF3, which may possibly modulate renal tubule fibrosis through TGF-β [149]. TGF-β generates intracellular signals through SMADs and is elevated in fibrosis models and chronic kidney disease [150]. Specifically, TGF-β plays a role in ECM deposition in the mesangium and perhaps in epithelial–mesenchymal transition (EMT) in tubular cells, through the exact role of TGF-β in EMT is under debate [8,151].

Exome sequencing, epigenomics, and transcriptomics have provided additional insights into the pathogenesis of CKD fibrosis. Homozygosity mapping of a family with karyomegalic interstitial fibrosis revealed a homozygous nonsense mutation in FAN1, a nuclease that repairs DNA inter-strand crosslinks, suggesting a connection between DNA repair and renal fibrosis [152]). Through epigenomics, DNA methylation changes have been implicated in aging kidney fibrosis of both the glomerulus and the interstitium [153]. In particular, DNA methylation can be specifically localized to CKD gene targets such as RASAL1, which also is found to be methylated in liver fibrosis [150]. A complementary comparison study between African American and Caucasian patients with CKD uncovered an association between lncRNA LINC00923 and CKD progression [154]. Transcriptome analysis of the aging kidneys in which nephrons scar and involute identified genetic variants in age-related genes including TSPYL5, which modulates p53 expression and telomerase activity [155]. Transcriptome data from micro-dissected kidney specimens with different clinical presentations of CKD identified a subset of genes enriched for immune signaling pathways whose expression correlated with fibrosis even after adjusting for functional indices like eGFR [156]. Another study profiled renal epithelial cells stimulated with TGF-β ex vivo and confirmed the resulting pro-fibrotic signature in human diabetic nephropathy tissue [157].

There has recently been an abundance of discoveries of differential miRNA expression in fibrotic tissues and may be a potential source of new therapies in the future through antimir oligonucleotides development. However, nearly all miRNA studies for renal fibrosis are limited to rodent models and are outside the scope of this review. It is worth noting that urinary sediment miRNAs can serve as non-invasive biomarkers of kidney disease, much like uromodulin. In a study of IgA nephropathy, miR-21 and mir-205 were significant biomarkers that could be used to monitor disease progression [158].

Approved anti-fibrotic therapies for CKD currently include drugs that modulate the renin-angiotensin-aldosterone system (RAAS), such as ACE inhibitors, ARBs, and statins which likely modulate TGF-β expression [159]. The central role of TGF-β in renal fibrosis has directed the focus of many clinical trials to TGF-β directed therapies. A humanized monoclonal TGF-β antibody was tested in clinical trials in patients with diabetic nephropathy, but was terminated early due to lack of efficacy [160]. Fresolimumab, which inhibits three isoforms of human TGF-β, did not progress beyond phase II clinical trials due to underpowering of studies [161,162]. Pirfenidone, a small molecule inhibitor of TGF-β signaling, improved GFR in a small study of focal segmental glomerulonephritis patients [163] as well as in a randomized double-blind placebo-controlled study in patients with diabetic nephropathy [163].

### 4.2. SLE and Lupus Nephritis

Beyond CKD, renal fibrosis often occurs in the setting of systemic lupus erythematosus (SLE) as a consequence of autoimmune inflammation leading to lupus nephritis. SLE is a complex, heterogenous autoimmune disease that develops from an interplay of genetic, environmental and immunologic risk factors [164] and can affect nearly every organ system. Microarray profiling of biopsied glomeruli from patients with lupus nephritis identified fibrotic biomarkers including COL1A2, COL6A3, MMP7, DSP, KRT18, and CCL2 [165]. Single cell RNA sequencing of renal biopsies showed elevated type I interferon in the tubular cells compared to controls, a marker that was also correlated to treatment resistance. In treatment-resistant patients, pathway analysis revealed the upregulation of ECM proteins TIMP1 and SERPING in renal tubular cells, both of which are associated with renal fibrosis. Interestingly, COL1A1, COL14A1, COL1A2, and COL5A2 expressions in renal tubules as well as in non-lesional patient skin biopsy keratinocytes could be used to differentiate between treatment responders and non-responders [166].

Several miRNAs have demonstrated utility as biomarkers in lupus nephritis. Tissue levels of miR-150, which is downstream of anti-fibrotic protein suppressor of cytokine signaling 1 (SOCS1), is a biomarker of disease chronicity [167]. MiR-3201 and miR-1273e were downregulated in lupus nephritis as compared with diabetes-associated nephrosclerosis [168]. MiR-21, miR-150, and miR423 were identified as circulating plasma biomarkers [169]. In urinary exosomes of patients with lupus nephritis, miR-146 served as a biomarker of disease-associated fibrosis in a mechanism involving TRAF6 signaling [170], while miR-21, miR0150, and miR-29c levels correlated with disease chronicity via their targeting of VEGFA and SP1 [171,172].

Regarding extra-renal manifestations of SLE, transcriptome profiling of cutaneous SLE tissue identified downregulation in miR-150, miR-21, and miR-1246 [173]. In discoid lesions, upregulation of miR-31 and miR-485-3p contributed to skin inflammation and fibrosis [174], which was linked to overexpression of TGF-β pathway genes SERPINE1, MMP9, and TGFBR1 and phosphorylation of SMAD3 and TGFB1 [175].

## 5. Lung Fibrosis

Idiopathic pulmonary fibrosis (IPF) is an incapacitating, fatal, progressive disease that represents the most common form of idiopathic interstitial pneumonia [176,177]. IPF is characterized by initial damage to the alveolar epithelium, which sends signals to fibroblasts and macrophages, stimulating tissue damage and synthesis of ECM. NETs released from neutrophils contribute to inflammation and further activate associated myofibroblasts to perpetuate fibrosis [11]. The resultant destruction of parenchyma and replacement of alveoli by dense fibrotic tissue causes decline in lung function, leading to respiratory failure and death [178]. Although the pathogenesis of IPF is not well understood, various environmental and infectious triggers as well as genetic risk factors are associated with its development. Among environmental factors, 20+ pack-years of smoking has a strong causative relationship. Other environmental exposures that increase IPF risk are metal dusts (brass, lead, and steel) and wood dust (pine). Occupational factors include farming, raising birds, hair dressing, stone cutting/polishing, and exposure to livestock and to vegetable dust/animal dust [179,180,181]. In addition, several viruses including Epstein-Barr virus (EBV) hepatitis C, cytomegalovirus, human herpesvirus (HHV-) 7, and HHV-8 may play a role in the pathogenesis of IPF [182,183,184,185].

A growing body of evidence in the form of GWAS have supported the role of genetic factors in the development of familial and sporadic IPF, to the extent that at least one-third of the risk for developing fibrotic IIP is explained by common genetic variants [186]. Risk loci in ELMOD2 (on chromosome 4q31) [187,188,189,190,191], SFTPA2 (encoding A2 surfactant protein) [192], and genetic variants within the human telomerase reverse transcriptase (hTERT) or RNA (hTR) components of the telomerase [193,194,195,196,197] have been associated with familial IPF. Other studies in sporadic IPF patient cohorts have identified polymorphisms in genes encoding cytokines IL-1 α, TNFα, lymphotoxin α, IL-4, IL-6, IL-8, IL-10, and IL-12 enzymes (α_1_-antitrypsin and angiotensin-converting enzyme) [198,199,200], profibrotic molecules TGFB1, coagulation pathway genes PAI1 and PAI2, genes for surfactant proteins A and B, immunomodulatory NOD2/CARD15, and matrix metalloproteinase MMP1 in association with increased risk of IPF. Human leukocyte antigen (HLA) class I and class II allele haplotypes have also been shown to have a skewed distribution among patients with IPF [201,202,203]. Additional IPF GWAS have identified independent association signals with genes linked to epithelial cell function, including lung defense (MUC5B), telomere maintenance (TERT and STN1), and cell–cell adhesion (DSP and DPP9) [204]. While telomerase mutations are associated with short telomeres and more rapid disease progression [205], MUC5B and toll-interacting protein (TOLLIP) variants are associated with slowed progression [206]. AKAP13 has been identified as a potential driver of IPF pathogenesis; rs62025270 was associated with disease susceptibility and correlated with increased AKAP13 expression in fibrotic lung tissue [207]. Taken together, genes in IPF-associated risk loci are implicated in a series of biological processes including alveolar stability, host defense, cell–cell barrier function, and cellular senescence [186].

Transcriptome profiling by microarray and next-generation sequencing has been used to identify differential gene expression in IPF, using genomic material from IPF lung and ex vivo fibroblasts isolated from IPF samples, in comparison with normal lung samples [208]. For instance, profiling of non-cultured IPF fibroblasts identified altered expression of genes involved in global processes of cellular homeostasis, including Wnt signaling, apoptosis, and cell cycle regulation [209]. Most recently, single-cell RNA-seq of lung parenchyma from IPF patients catalogued the spectrum of cell populations, which included myofibroblasts, profibrotic macrophages, and a new population of epithelial basaloid cells expressing mesenchymal markers that neighbor myofibroblast collections in the lung [210].

Non-coding RNAs also have demonstrated roles in IPF pathogenesis. MiRNA profiling of lung tissue from IPF patients has been performed by numerous groups, though functional implications of differential miRNA expression in IPF has primarily been studied in animal models. Pro-fibrotic miR-21 and miR-155 expressions are differentially upregulated and anti-fibrotic let-7a, miR-29, miR-30, and miR-101 expressions are differentially downregulated in IPF patient lungs [211,212,213,214,215]. The expression of miR-21, which downregulates SMAD7 leading to increased pro-fibrotic canonical TGFβ pathway signaling, was increased in the serum of IPF patients, and its levels correlated with a decrease in lung function [216]. Njock et al. [217] performed miRNA expression profiling of sputum-derived exosomes from IPF patients, identifying 21 miRNAs that were differentially expressed, including upregulated miR-142-3p and miR-33a-5p and downregulated let-7d-5p. In addition to miRNAs, long non-coding RNAs are emerging as contributors to IPF development (reviewed in [218]); as one example, lncITPF is highly upregulated in lung fibrosis [219].

Advances in therapies have led to the development and approval of two drugs that slow the rate of fibrosis and associated functional decline in IPF: nintedanib, a tyrosine kinase inhibitor that targets growth factor signaling downstream of VEGFR, FGFR, and PDGFR; pirfenidone, which exerts anti-inflammatory as well as anti-fibrotic effects via inhibition of collagen synthesis, downregulation of TGF-β and TNF alpha, and decreased fibroblast proliferation [177]. Proteomic analysis of serum from IPF patients at baseline and 1 year after nintedanib treatment identified differences in proteins related to cell differentiation and epithelial-mesenchymal transition [220]. Pamrevlumab, an antibody therapy targeting connective tissue growth factor, is currently in phase II clinical trials.

## 6. Cardiac Fibrosis

The heart has minimal regenerative potential as compared to other organs. Multivariate factors influence the development of cardiac fibrosis, including diabetes, systemic hypertension, ischemia, and primary cardiomyopathies [221]. Cardiac fibrosis is initially beneficial in the setting of myocardial infarction or other cardiac insults by preventing heart wall rupture, but prolonged fibroblast activation leads to fibrosis beyond the infarcted tissue. The excessive ECM deposition causes ventricle stiffness, decreased compliance, and eventual heart failure [222]. Despite the major health burden of cardiac fibrosis, there are limited antifibrotic therapies available, and there is a need for studies to elucidate the factors driving fibrosis in cardiac disease [221].

RNA-Seq of cardiac tissue from patients with dilated cardiomyopathy identified differential expression of WWP2, an E3 ubiquitin protein ligase 2, which regulates SMAD function and subsequent development of cardiac fibrosis [223]. Transcriptome analysis of 15 patients with ischemic cardiomyopathy identified 35 lncRNAs involved in the regulation of extracellular matrix proteins [224]. In another study, gene expression profiling identified CORIN, FIGF, and COL1A1 as playing putative roles in fibrosis of cardiomyopathy [225]. Another study [226] used machine learning to analyze RNA-seq data in patients with dilated and ischemic cardiomyopathy for the purposes of improving diagnostic accuracy, identifying a series of “highly contributing genes” relating to fibrosis that achieved 93% accuracy. Additionally, epigenomic studies of dilated cardiomyopathy patients found that abnormal DNA methylation changed mRNA expression of LY75 and ADORA2A [227] with later studies linking 517 loci with dilated cardiomyopathy [228]. A case-control GWAS identified a SNP within contractile protein-encoding FHOD3 with risk of hypertrophic cardiomyopathy [229].

Other genomic studies in fibrosing cardiac disorders have focused on changes in gene expression and regulation in the context of myocardial infarction. GWAS identified SNPs within the novel gene MIAT as conferring risk for myocardial infarct [230]. Through microarray analysis, galectin-3 was reported to be overexpressed in infarcted myocardium, which was clinically verified in patients with ST-elevation myocardial infarction [231]. Biopsies of infarcted tissues in transplanted hearts showed upregulation of pro-fibrotic miR-21, miR-214, and miR-223 and downregulation of anti-fibrotic miR-29b and miR-149 [232]. Transcriptomic analysis of circulating plasma RNA in post-myocardial infarction patients found mitochondrial long noncoding RNA uc022bqs.1 (LIPCAR) to be differentially regulated depending on the stage of disease progression, serving as a predictor of future death in patients with heart failure [233]. Moreover, ex vivo stimulation of differentiated human cardiac fibroblasts with TGF-β followed by RNA-seq analysis identified a critical role for upregulation of interleukin-11 (IL11) in cardiac fibrosis [234].

With regard to therapeutics, clinical trials have been designed to target molecules implicated in fibroblast activation (TGF-β, endothelin, Angiotensin II, CCN2, PDGF, and others), as activated fibroblasts increase expression of alpha-smooth muscle actin to increase ECM deposition; however, most studies are still in the pre-clinical phase [235]. The few approved cardiac antifibrotic therapies modulate the renin-angiotensin-aldosterone system, such as angiotensin receptor inhibitors, which has been shown to slow cardiac fibrosis but not completely inhibit or reverse its progression [236]. Relaxin, a vasodilatory peptide hormone expressed in the heart, endometrium, mammary gland, placenta, and prostate has been reported to suppress cardiac remodeling [237], and human recombinant serelaxin inhibits production and perhaps even mediates the reversal of fibrosis through relaxin family peptide receptor 1 (RXFP1) [237]. Unfortunately, while initial trials were promising, phase III trials of serelaxin showed no significant difference in worsening heart failure or incidence of death in the treatment group vs. placebo [238].

## 7. Conclusions and Future Directions

**Figure 1:**
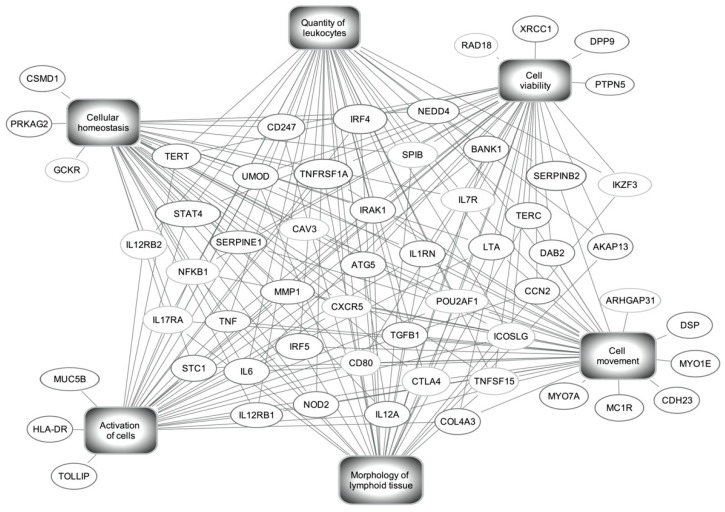
GWAS of fibrosing disorders. Pooled network of genes from genome-wide association studies (GWAS) of fibrotic diseases in skin, liver, lung, kidney, and heart, and their associated biological processes. Figure created using Ingenuity Pathway Analysis (IPA) Summer Release (June 2020).

In this review, we have synthesized findings from a diverse spectrum of genomic studies conducted in patient samples across multiple platforms. Findings from these studies have elucidated the pathophysiologic mechanisms that underlie the development of fibrosis in multiple organs. While the inciting acute injury trigger may vary, fibrosis is the shared end result of a disordered wound healing response. It is, therefore, not surprising that many of the genes and pathways reported in the studies herein are conserved among different tissues (Figure 1). Ultimately, the design and development of therapies targeting shared and distinct processes of fibrosis identified through genomic studies will result in improved quality of life for millions of patients suffering from fibrotic diseases.

## Figures and Tables

**Table 1:** Wounding as a trigger of human fibrosing disorders in multiple organs.

Organ	Injury Trigger	Fibrotic Disease (s)
Skin	Breach of epidermal barrier	Keloid; hypertrophic scar; chronic ulcer
	Vascular endothelial cell injury	Scleroderma/SSc ^1^; morphea; lichen sclerosus
	Exogenous insult (radiotherapy, heat, contrast agent)	Radiation fibrosis; burn; nephrogenic systemic fibrosis
Liver	Exogenous insult (alcohol), infection, cholestasis, NASH ^2^	Cirrhosis (hepatic cirrhosis; primary biliary cirrhosis)
Kidney	Obstruction, infection, autoimmune destruction, underlying disease (diabetes, hypertension)	Chronic kidney disease; SLE ^3^
Lung	Alveolar epithelial damage	Idiopathic pulmonary fibrosis
Heart	Genetic defect, ischemia	Cardiomyopathy; heart failure

^1^ SSc, systemic sclerosis. ^2^ NASH, non-alcoholic steatohepatitis. ^3^ SLE, Systemic Lupus Erythematosus.

## Abbreviations

ACE	Angiotensin-converting enzyme
ADORA2A	Adenosine A2a receptor
AFF3	AF4FMR2 family member 3
AGAP2	ArfGAP With GTPase Domain, Ankyrin Repeat And PH Domain 2
AGES	Age, gene/environment susceptibility
ALMS1	Alstrom syndrome 1
ARB	Angiotensin II receptor blocker
ARHGAP31	Rho GTPase activating protein 31
ATG5	Autophagy related 5
ATXN2	Ataxin 2
BANK1	B Cell Scaffold Protein With Ankyrin Repeats 1
BCAS3	Breast carcinoma amplified sequence 3
BLCC	Bioengineered bilayered living cell construct
CACNA1G-AS1	CACNA1G antisense RNA 1
CAV1	Caveolin-1
CCN2	Cellular communication network factor 2
CD80	Cluster of differentiation 80
CDH1	Cadherin 1
CDH23	Cadherin 23
CDK4	Cyclin-dependent kinase 4
circRNA	circular RNA
CKD	Chronic kidney disease
COL1A1	Collagen type I alpha 1
COL4A3	Collagen type IV alpha 3
COL5A1	Collagen type V alpha 1
CORIN	Corin, atrial natriuretic peptide-converting enzyme
CPS1	Carbamyl phosphate synthetase I
CSF1R	Colony stimulating factor 1 receptor
CSMD1	CUB And Sushi Multiple Domains 1
CTBP1	C-terminal binding protein 1
CTGF	Connective tissue growth factor
CTLA4	Cytotoxic T-lymphocyte associated protein 4
CTSS	Cathepsin S
CTSZ	Cathepsin Z
CXCL10	C-X-C motif chemokine ligand 10
CXCR5	C-x-c motif chemokine receptor 5
DAB2	Disabled homolog 2
DACH1	Dachshund homolog 1
DGKA	diacylglycerol kinase alpha
DPP9	Toll-interacting protein
EBV	Epstein Barr virus
ECM	Extracellular matrix
EGR1	Early Growth Response 1
eMERGE	Electronic medical records and genomics
EMT	Epithelial-mesenchymal transition
eQTL	Expression of quantitative trait
FAN1	Fanconi anemia associated nuclease 1
FGFR	Fibroblast Growth Factor Receptor
FIGF	C-fos–induced growth factor
FKBP10	FK506-binding protein 10
FN1	Fibronectin 1
FOXL2	Forkhead box L2
GABRR1	Gamma-aminobutyric acid type A receptor subunit rho1
GALNT11	Polypeptide N-acetylgalactosaminyltransferase 11
GCKR	Glucokinase regulator
GRB10	Growth factor receptor bound protein 10
GWAS	Genome-wide association study
HBV	Hepatitis B virus
HCV	Hepatitis C virus
HGF	Hepatocyte growth factor
HHV	Human herpesvirus
HIF-1α	Hypoxia inducible factors
HLA	Human leukocyte antigen
HLA-DQB1	Major histocompatibility complex, class II, DQ beta 1
HNF1A-AS1	HNF1A Antisense RNA 1
HSC	Hepatic stellate cells
HSD17B13	Hydroxysteroid 17-beta dehydrogenase 13
hTERT	Human Telomerase Reverse Transcriptase
hTR	Human Telomerase RNA
ICOSLG	Inducible T-cell costimulator ligand
IGF, IGFBP	Insulin-like growth factor, Insulin-like growth factor binding protein
IIP	Idiopathic interstitial pneumonia
IKZF3	IKAROS family zinc finger 3
IL11	Interleukin-11
IL12A	Interleukin-12 subunit alpha
IL12B	Interleukin-12 subunit beta
IL12RB2	Interleukin 12 receptor subunit beta 2
IL17RA	Interleukin 17 receptor A
IL7R	Interleukin 7 receptor
IPF	Idiopathic pulmonary fibrosis
IRAK1	Interleukin 1 receptor associated kinase 1
IRF4, 5, 7	Interferon regulatory factor
IRF5-TNPO3	Interferon regulatory factor 5 - transportin 3
ITGB2	Integrin subunit beta 2
LAPTM5	Lysosomal protein transmembrane 5
LASS2	Ceramide synthase 2
LINC00923	Long intergenic non-protein coding RNA 923
LIPCAR	Long non-coding cardiac associated RNA
lncRNA	Long non-coding RNA
LY75	Lymphocyte antigen 75
LYPLAL1	Lysophospholipase like 1
MAPK	mitogen-activated protein kinase
MIAT	Myocardial infarction associated transcript
miRNA	MicroRNA
MMP 1	Matrix metallopeptidase 1
MMP2	Matrix metalloproteinase 2
MMP9	Matrix metallopeptidase 9
MYO 1E, MYO7	Myosin 1E, Myosin 7
NAFLD	Non-alcoholic fatty liver disease
NCAN	Neurocan
NEDD4	Neural precursor cell expressed developmentally down-regulated protein 4
NET	Neutrophil extracellular trap
NFKB1	Nuclear factor kappa B subunit 1
NOD2/CARD15	Nucleotide-binding oligomerization domain-containing protein 2 / Caspase Recruitment Domain-Containing Protein 15
NOTCH4	Neurogenic locus notch homolog 4
OTUD6B	Ovarian tumor domain deubiquitinase 6B
PAI1/ PAI2	Plasminogen Activator Inhibitor 1/ Plasminogen Activator Inhibitor 2
PBC	Primary biliary cirrhosis
PDGF	Platelet-derived growth factor
PDGFR	Platelet Derived Growth Factor Receptor
PIP5K1B	Phosphatidylinositol-4-phosphate 5-kinase type 1 beta
PLEK	Pleckstrin
PNPLA3	Patatin like phospholipase domain containing 3
POGLUT1	Protein O-glucosyltransferase 1
POLD1	DNA Polymerase Delta 1, Catalytic Subunit
POU2AF1	POU domain class 2-associating factor 1
PRKAG2	Protein kinase AMP-activated non-catalytic subunit gamma 2
PTPN5	Protein tyrosine phosphatase non-receptor type 5
PYGO1	Pygopus family PHD finger 1
RAAS	Renin-angiotensin-aldosterone system
RASAL1	RAS protein activator like 1
RUNX2	Runt-related transcription factor 2
RXFP1	Relaxin family peptide receptor 1
SAMM50	Sorting and assembly machinery component 50 homolog
SFTPA2	surfactant protein A2
SHROOM3	Shroom family member 3
SLC22A2	Solute carrier family 22 member 2
SLC34A1	Solute carrier family 34 member 1
SLC6A13	Solute carrier family 6 member 13
SLC7A9	Solute carrier family 7 member 9
SLE	Systemic lupus erythematosus
α-SMA	Alpha smooth muscle actin
SOCS1	Suppressor of cytokine signaling 1
SOX9	SRY-box transcription factor 9
SPIB	Spi-b transcription factor
SPP1	Secreted phosphoprotein 1
SSc	Systemic sclerosis
STAT4	Signal transducer and activator of transcription
STC1	Stanniocalcin 1
TFDP2	Transcription factor DP-2
TGF-β	Transforming growth factor beta
TIMMDC1	Translocase of inner mitochondrial membrane domain containing 1
TM6SF2	Transmembrane 6 superfamily member 2
TMEM39A	Transmembrane protein 39a
TMEM60	Transmembrane protein 60
TNC	Tenascin C
TNFRSF1A	TNF receptor superfamily member 1a
TNFSF15	TNF superfamily member 15
TOLLIP	Matrix Metalloproteinase
TRAPPC9	Trafficking protein particle complex 9
TSIX	Antisense to Xist (X-inactive specific transcript)
TSPYL5	Testis-specific Y-encoded-like protein 5
TXNRD2	thioredoxin reductase 2
TYROBP	TYRO protein tyrosine kinase-binding protein
UBE2Q2	Ubiquitin conjugating enzyme E2 Q2
UMOD	Uromodulin
VEGFA	Vascular endothelial growth factor A
VEGFR	Vascular Endothelial Growth Factor Receptor
VLU	Venous leg ulcer
WDR37	WD repeat domain 37
WDR72	WD repeat domain 72
WWP2	WW domain-containing protein 2
XRCC1	X-Ray Repair Cross Complementing 1
ZFP90	Zinc finger protein 90 homolog
